# Friday Night Is Pizza Night: A Comparison of Children’s Dietary Intake and Maternal Perceptions and Feeding Goals on Weekdays and Weekends

**DOI:** 10.3390/ijerph15040720

**Published:** 2018-04-11

**Authors:** Debra A. Hoffmann, Jenna M. Marx, Jacob M. Burmeister, Dara R. Musher-Eizenman

**Affiliations:** 1 Department of Psychology, Bowling Green State University, Bowling Green, OH 43403, USA; 2 Department of Psychology, University of Findlay, Findlay, OH 45840, USA

**Keywords:** school-age children, weekday, weekend, dietary intake, portion size, food perceptions, feeding goals

## Abstract

Childhood obesity is a serious issue in the U.S. While obesity is the result of a multitude of factors, a great deal of research has focused on children’s dietary intake. While children’s eating patterns vary throughout the week, not much else is known about weekday-weekend differences. Therefore, the current study examined differences in the frequency and portion size of school-age children’s consumption of common foods and beverages, as well as mothers’ perceptions of those items and their child feeding goals, on weekdays and weekends. A total of 192 mothers of children aged 7 to 11 were recruited through Amazon’s Mechanical Turk. Results showed a consistent pattern of more frequent consumption and larger portions of unhealthy foods and beverages on weekends. This aligned with mothers’ perceptions of those foods and beverages as weekend items, as well as their feeding goals of health and price being less important on weekends. It is quite possible that weekends are viewed as having less structure and facilitate schedules that allow children to consume more meals away from home. These findings shed light on additional risk factors in children’s eating patterns and highlight the serious implications that day of the week can have on childhood obesity.

## 1. Introduction

Approximately one-third of school-age children are overweight or obese (Body Mass Index (BMI) ≥ 85th percentile), of which over half are obese [1,2]. Despite efforts to address this [3,4,5], obesity has increased by more than 60% amongst this age group over the last three decades [6]. This is problematic because not only can obesity persist into adulthood [7,8], but childhood obesity is associated with a number of risk factors, such as hypertension and insulin resistance, which can lead to more detrimental chronic diseases [9,10]. While obesity is the result of a complex interaction between environmental, behavioral, and genetic factors [11], a great deal of research has focused on children’s dietary intake, as the consumption of unhealthy, or energy-dense, low-nutrient foods and beverages have been linked to higher BMI [12,13,14].

Parents play an integral role in shaping children’s eating behaviors. For example, they introduce their children to new foods, determine what foods are available and how accessible they are in the household, establish family mealtime practices, such as eating together at the dining table or watching TV while eating, model eating-related attitudes and behaviors, and use child feeding practices (e.g., restriction, pressuring, and giving food as a reward). Parents’ engagement in these practices communicates explicitly and implicitly to the child their own values, expectations, and goals, which influences children’s dietary preferences, energy intake, relationship with food, and weight status [15,16,17,18,19]. Because of their role as gatekeepers to their children’s food environment, parents are often targets for prevention and intervention programs.

In addition to variability from one family to another, there is also variability within families due to contextual factors. For example, the day of the week can influence children’s consumption. Although this is a relatively sparse area of research to-date, it appears that the quality of children’s diet declines on weekends compared to weekdays [20,21,22,23,24,25]. For example, Rothausen et al. (2012) found that four- to ten-year-olds had higher intakes of sweets, consumed a greater percentage of energy from added sugars, had higher overall energy intake, and had lower fiber and fruit and vegetable intake on weekends than on weekdays [21]. These findings were consistent with another study that found that children aged six to nine had higher intakes of total sugars and foods and beverages high in added sugar on weekends than on weekdays [23]. Moreover, Cullen et al. (2002) reported that fourth to sixth-grade children engaged in more high-fat practices (e.g., chose high-fat foods or added fat to foods), used fewer low-fat practices, and consumed a higher percentage of energy from fat on weekends relative to weekdays [20]. Though this relationship has been more consistently observed in European than American samples, the overall trend remains; children tend to eat less healthy on the weekends.

This is concerning because health habits developed in childhood can persist into and impact later adulthood, and can be increasingly difficult to change, especially as those habits become more engrained [26,27,28,29]. In fact, these weekday-weekend dietary differences are present in adults as well. Not only do adults consume significantly more calories on weekends, but they also exercise less [30,31,32,33,34]. This unhealthy weekend lifestyle pattern contributes to weight gain and can even be detrimental to weight loss [31,34]. As habits are developed during childhood and maintained over time [35], it is critically important to understand more about how these eating patterns are established and factors that contribute to these differences.

While we know that children’s eating patterns vary throughout the week, not much else is known about weekday-weekend differences. For example, previous research has examined differences on a broader scale (e.g., identifying certain food groups, such as added sugars), but less is known about what specific foods children are consuming and whether any foods are more problematic or concerning. Additionally, it is unclear whether children are consuming a particular food more frequently on weekends or if they are consuming more of that item when they have it (i.e., larger portions). Understanding this distinction between frequency and portion sizes can enhance treatment efforts by identifying more concrete areas that can be addressed. Only one study that we are aware of has examined differences in portion sizes across the week, albeit with adults [36]. In that study, meals consumed on weekends were found to be 12% larger than on weekdays. However, that study was conducted almost thirty years ago and portion sizes have significantly increased since then, even exceeding federal standards [37,38]. And, since parents are pivotal in shaping their children’s food environment, it is quite possible that children may be consuming larger portion sizes on weekends as well. This would be particularly concerning, since when given larger portion sizes, children consume larger amounts (by as much as 75%) [39], resulting in excess energy intake, and do not appear to sufficiently compensate for this at subsequent meals [40].

Another question is why children are eating differently on the weekends relative to weekdays. Is it possible that parents are more willing to “bend the rules” when it comes to their children’s eating because it is the weekend? This makes sense, given that parents already make exceptions to these rules on special occasions or under certain circumstances (e.g., when it is hot outside or their child is sick) [41]. However, no studies have examined parental feeding goals in the context of the day of the week. Research indicates that parents value health and practicality [42,43,44] and establish food rules or engage in certain child feeding practices based on those values (e.g., restricting, pressuring, or providing food as a reward to increase or reduce children’s intake of certain foods) [41,45]. In fact, these values influence how parents feed their children, with those more motivated by health feeding their child nutrient-dense foods and those motivated by practicality (e.g., price and convenience) feeding their child energy-dense foods [46,47]. It is also possible that parents perceive certain foods and beverages as more of a weekday or weekend item, which could influence its availability and consumption in the household. It has already been shown that parents make food purchasing decisions for their child based on their perceptions of the qualities of a food product (e.g., nutrient claims, cost, and their child’s taste preferences) [44,48,49]. As such, understanding these relationships could provide new insight in this area and can have important and serious implications on the importance of contextual factors in child feeding.

Therefore, the current study sought to build and expand on previous research by examining whether school-age children’s consumption of common foods and beverages, along with their portion sizes, differed on weekdays relative to weekends. Mothers were also asked about their perceptions of these foods and beverages, as well as their feeding goals, to explore whether differences existed across the week.

## 2. Materials and Methods

### 2.1. Sample

One hundred ninety-two mothers of children age 7 to 11 (*M*_age_ = 8.66, *SD* = 1.4) were recruited through Amazon’s Mechanical Turk, an online workforce community, which has been shown to be a reliable source of data collection [50,51,52,53]. Participants were eligible for the study if they were the mother and primary caretaker of a child between the ages of 7 to 11, lived in a two-parent household, were fluent in English, and lived in the United States. Two quality control questions (e.g., “Please select disagree”) were included in the survey to ensure that participants were attentive and providing quality responses. All subjects gave their informed consent for inclusion before they participated in the study. The study was conducted in accordance with the Declaration of Helsinki and the protocol was approved by the Ethics Committee of Bowling Green State University (project identification code 423547).

Mothers had a mean age of 34.2 (*SD* = 6.8), were predominately Caucasian (76%) and had at least some college education (88%). Almost half (43%) were employed full- or part-time and 52% had an annual household income above $55,000. Mothers’ mean BMI was 27.0 (*SD* = 7.3, range = 15.3–63.1) and about half (49%) were classified as overweight or obese (BMI ≥ 25).

Data collection was limited to only mothers because they are still typically considered the primary caregiver and decision maker in child-care activities, including feeding [54,55]. We selected 7 to 11-year-olds because we were interested in elementary school-age and two-parent households to ensure more consistency in children’s eating routines, as research suggests that there are differences in the eating habits of children in single- versus two-parent households [56,57].

### 2.2. Measures

Participants were presented with a list of common foods and beverages that covered a wide spectrum of nutritional values (see Table 1) and asked questions about them. These items were selected because of their use in previous dietary research [58,59,60,61,62]. Participants reported how often their child consumed each item on a 5-point scale, ranging from 1 (never/rarely) to 5 (almost every day). The scale was slightly modified from another study [63] to ask specifically about weekday and weekend eating. In this study, weekday was defined as Monday through Friday afternoon and weekend as Friday evening through Sunday. Internal consistency scores were similar to the reference study, with Cronbach’s alphas of 0.69 and 0.76 for weekday healthy (i.e., fruits, vegetables, whole grains, and water) and unhealthy (i.e., chips, fast food, fried food, pizza, sweet snacks/desserts, and soda) items, and 0.72 and 0.75 for weekend items, respectively. Participants were also asked whether they perceived each food and beverage as more of a weekday or weekend food and whether their child consumed larger portions of each item on weekdays or weekends.

Additionally, two subscales (health and price) from the Food Choice Questionnaire [64] were included to assess parental feeding goals. Participants rated the importance of each item (health: 4 items, e.g., “It is important to me that the foods my child eats are nutritious”; price: 3 items, e.g., “It is important to me that the foods my child eats are good value for the money”) for weekdays and weekends on a 5-point scale, from 1 (not at all) to 5 (completely). These subscales were selected because of their importance in food selection and consumption [42,65,66,67,68]. Cronbach’s alphas for the price subscale were 0.83 for weekday and 0.84 for weekend and, for health, were 0.84 for each. These ranges were consistent with the validation study sample [64].

### 2.3. Data Analysis

Descriptive statistics were used to describe the sample. Preliminary analyses of bivariate correlations, ANOVAs, chi-squares, and independent samples *t*-tests examined whether demographic variables were related to the variables of interest (i.e., weekday-weekend differences in dietary intake, portion sizes, feeding goals, and perceptions). No significant relationships were observed, so no covariates were controlled for in subsequent analyses. To compare weekday-weekend differences, paired samples *t*-tests were used to analyze dietary intake and feeding goals, and nonparametric chi-square tests analyzed portion sizes and perceptions of each food and beverage item. The Benjamini-Hochberg correction [69] was used to control for multiple tests. All analyses were completed using SPSS version 20 (IBM, Armonk, NY, USA).

## 3. Results

### 3.1. Food Environment

Nearly all mothers reported that they make the primary feeding-related decisions for their child compared to their spouse or partner (weekdays: 88% vs. 9%; weekends: 83% vs. 11%). About half (52%) of mothers reported that their child helps themselves to food on their own (e.g., take food out of the fridge/pantry and buy food from the store/vending machine). On weekdays, 32%, 19%, and 9% of mothers reported that their child helps themselves often or always to food during breakfast, lunch, and dinner, while 19% and 62% did for desserts and snacks. On weekends, these frequencies were 37%, 26%, and 12% during breakfast, lunch, and dinner, with 23% and 61% for desserts and snacks. No significant differences were found for these frequencies between weekday and weekend. An overwhelming majority of mothers reported that they did not have certain mealtime practices or food rules that they only enforce on weekdays or weekends (86% and 94%, respectively).

### 3.2. Dietary Intake

Mothers reported that their child more frequently consumed vegetables (*t*(191) = 2.80, *p* < 0.01), whole grains (*t*(191) = 2.16, *p* < 0.05), and water (*t*(191) = 2.65, *p* < 0.01) on weekdays, and chips (*t*(191) = −4.78, *p* < 0.001), fast food (*t*(191) = −7.31, *p* < 0.001), fried food (*t*(191) = −5.67, *p* < 0.001), pizza (*t*(191) = −5.18, *p* < 0.001), sweet snacks/desserts (*t*(191) = −4.78, *p* < 0.001), and soda/pop (*t*(191) = −3.76, *p* < 0.001) on weekends. See Table 1.

### 3.3. Portion Sizes

Mothers reported that, on weekends, their child consumed larger portions of soda/pop (weekday: 3% vs. weekend: 51%, *p* < 0.001), sweet snacks/desserts (3% vs. 49%, *p* < 0.001), chips (4% vs. 44%, *p* < 0.001), pizza (6% vs. 47%, *p* < 0.001), fried food (6% vs. 41%, *p* < 0.001), and fast food (9% vs. 53%, *p* < 0.001); whereas, on weekdays, they had larger portions of vegetables (18% vs. 5%, *p* < 0.001) and whole grains (13% vs. 5%, *p* < 0.05). See Table 2.

### 3.4. Maternal Perceptions

Mothers were more likely to perceive soda/pop (weekday: 4% vs. weekend: 56%, *p* < 0.001), chips (3% vs. 40%, *p* < 0.001), sweet snacks/desserts (5% vs. 42%, *p* < 0.001), pizza (7% vs. 53%, *p* < 0.001), fried food (6% vs. 45%, *p* < 0.001), and fast food (10% vs. 56%, *p* < 0.001) as weekend items. No foods or beverages were classified as more of a weekday item. See Table 3.

### 3.5. Feeding Goals

Mothers placed a higher importance on both health (*t*(191) = 3.45, *p* < 0.001) and price (*t*(191) = 2.96, *p* < 0.01) on weekdays than weekends. See Table 4.

## 4. Discussion

The present study examined differences in the frequency and portion size of school-age children’s consumption of common foods and beverages, as well as mothers’ perceptions of those items and their child feeding goals, on weekdays and weekends. Mothers reported that their child more frequently consumed healthier foods and beverages (i.e., vegetables, whole grains, and water) on weekdays and unhealthier foods and beverages (i.e., chips, fast food, fried food, pizza, desserts, and soda) on weekends. Additionally, mothers reported that their child consumed larger portions of the unhealthy items on weekends and the healthy items, except for water, on weekdays. These consumption patterns of unhealthy foods and beverages were consistent with mothers’ perceptions of them as weekend items and health and price feeding goals being less important on weekends. 

This pattern observed in children’s dietary intake is in accordance with previous research in this age group that the quality of children’s diets declines on weekends [20,21,22,23]. The notable difference here is that fruit consumption did not vary between weekdays and weekends, as Hart et al. and Rothausen et al. had found [21,22]. This might be due to sample characteristics. Hart et al.’s sample consisted of treatment-seeking, overweight and obese children [22], and Rothausen et al.’s sample was recruited from European countries [21]. It is possible, for example, that the treatment seeking families made a more deliberate effort to serve fruits and vegetables on weekdays. It is also possible that there are differences between European and American children’s diets, as differences are found in adults’ diets [70,71] and child feeding practices [72,73].

One notable finding is that although mothers reported that there were differences in their child’s food consumption across the week, an overwhelming majority denied having rules that they only enforced on weekdays (86%) or weekends (94%). It may be that while mothers are aware that there are differences in their child’s eating, they do not consider it to be changing their food rules on weekends. For example, it is possible that these weekday-weekend differences are due to eating out more often, but that is not seen as breaking any rules. Another possibility is that parents’ food rules are less rigid, such that they may relax them a bit on the weekend, but not completely drop them. For example, parents acknowledge having rules for healthy and unhealthy foods, but make exceptions for special occasions or under certain circumstances, such as if the child behaved well [41,74]. In fact, one qualitative study found that some families allowed their children to decide on the menu one day each weekend [74].

Alternatively, mothers may deny having different food rules across the week because there really are none. Instead, it could be that mothers are not fully aware of the foods their child consumes on weekdays, as there are likely differences in the foods available at school and home. That is, while children generally have breakfast and dinner with their family on weekdays, mothers may not be as aware of what their child consumes for lunch or any snacks they have at school. This could be particularly true for older children who have more autonomy. With that said, when nine-year-olds were asked who the primary decision maker is on what foods they typically consume at school, 92% stated that their parents chose or provided them options, while only 3% indicated that they controlled what they consumed for lunch [75]. Perhaps surprisingly, in this study, almost 90% of meals and snacks on weekdays were reported to be controlled by parents. This could explain why parents are still considered accurate reporters for this age group, despite potential bias and misreporting (for a review, see [76]). 

Future research might interview children to corroborate parental reports of dietary intake and explore whether there are differences in children’s perceptions of weekday-weekend foods and rules (e.g., whether they have more access to certain foods on weekends). In addition, investigating the role of using food as a reward and how this may impact parental feeding practices could be an interesting area of study, particularly if there are differences in parental feeding practices on weekdays and weekends. For example, perhaps a child who has completed their chores during the week is rewarded with ice cream on Friday night and parents do not view this as a deviation from their typical feeding practices, as it is a structured part of their child’s life. This could be especially relevant, as, in one study, restriction of unhealthy foods was the most reported feeding practice on weekdays, whereas, on weekends, including children in dinner preparation was the most common [74]. Further investigation into how parental feeding practices and behavioral management practices intersect would significantly add to the current literature.

Regarding portion sizes, it is notable that parents reported larger portions of unhealthy foods and smaller portions of healthy foods for their child on weekends than weekdays. This is not surprising, however, given that children have a preference for larger portions of unhealthy foods and smaller portions of healthy foods [77]. Coupled with the fact that children spend more time in sedentary activities and engage in lower levels of physical activity on weekends [22,78,79], this can further exacerbate the energy imbalance, leading to weight gain [80,81,82]. However, it is unclear how large these portions are, in an absolute sense, as mothers only reported in relative terms (i.e., they compared their own child’s portion sizes, not to any standard or other children). Parents tend to report a lack of knowledge and awareness when it comes to appropriate portion sizes for their children [83,84]. It would be instructive for future research to ask parents to compare children’s weekday and weekend portion sizes using standardized reference points (e.g., a piece the size of a deck of cards or 1/8 of a 12” pizza) to better understand how larger portion sizes might be implicated in higher weekend calorie intake.

A possible explanation for the pattern of findings demonstrated here is that parents view the weekend as a time to let loose and relax, as these are typically days off of work and school, and thus have less structure. Weekends might also be filled with family activities (e.g., visiting extended family, playdates with friends, and sporting events). Because of this, it is quite possible that weekends facilitate schedules that allow children to consume more meals away from home, which is associated with higher caloric consumption and poorer diet quality [85,86]. Further support for this explanation is that mothers reported that both health and price were less important values when it comes to their child’s food intake on weekends, which would again align with eating out more on weekends. Additionally, if weekends are perceived in this way, that could explain why mothers classified the unhealthy foods and beverages as more of weekend as opposed to weekday items. That is, weekends might be associated with having foods that one enjoys or can indulge in (e.g., WHOA, or “forbidden,” foods that should only be consumed occasionally [87]). Furthermore, all of the unhealthy foods and beverages that were perceived as weekend items are generally considered convenient (e.g., pre-packaged/prepared) and can be eaten outside the home. Of note, because the data are cross-sectional, it is not clear, for example, whether perceptions of foods and beverages are influencing consumption or vice versa. Future research could examine this question experimentally by introducing a novel food as either a weekday or weekend item and testing the impact of this perception on patterns of consumption.

One important contribution of this research is that food categories that ranged in nutritional value were examined instead of broad food groups or macronutrient intake. An advantage of this approach is that it provides an indication of the specific contents of children’s diets and allows for more targeted and focused efforts that can be incorporated into interventions. For example, by focusing on specific food and beverage items that are problematic, a concrete target is identified that may be practical and accessible for parents to address, instead of providing broader, general guidelines that can be confusing and harder to follow (e.g., the percentage of energy from carbohydrates being higher on weekends). Further, this study highlights the role of contextual factors (in this case, day of the week) in child food consumption and how it may influence or even override parental values in child feeding.

While this study investigated a largely unexplored area of research, there are several limitations that should be noted. The sample was largely homogeneous, as it only included mothers in a two-parent household, who were predominantly college educated and Caucasian, and thus limits the generalizability of the findings. Additionally, there may have been other factors not assessed, such as the type of employment of the primary and secondary caregivers, job demands and flexibility, and extracurricular or community involvement of the child, that may have influenced children’s eating behaviors. Further, while mothers overwhelmingly reported that they were the primary decision-maker for feeding their child, it is still possible that the other caregiver may have had some shared feeding responsibilities as well and held different views on weekday and weekend foods and beverages. This study asked mothers to report on their child’s dietary intake, but it would be beneficial for future research to more directly observe eating behaviors or to use techniques, such as ecological momentary assessment, that is less subject to bias and misrepresentation [88]. This would also be useful in cases where mothers were not fully aware of what their child actually consumed, especially if they ate meals outside the home or when not under their mothers’ care.

## 5. Conclusions

Among the 20 most populous countries in the world, childhood obesity is highest in the U.S. [89]. Despite efforts to combat this, obesity has continued to increase among children [6]. Given that more than a quarter of each week is the weekend, understanding weekday-weekend differences in school-age children’s dietary intake, as well as mothers’ perceptions of common foods and beverages and their child feeding goals, may be a very important aspect of pinpointing the causes and possible solutions for this epidemic. Results showed a consistent pattern of more frequent consumption and larger portions of unhealthy foods and beverages on weekends. These weekend consumption patterns aligned with mothers’ perceptions of those unhealthy foods and beverages as being weekend items, as well as their goals of health and price being less important on weekends. These findings shed light on additional risk factors in children’s eating patterns that can be readily incorporated into interventions and, ultimately, highlight how the day of the week can be an important factor with serious implications in childhood weight problems and obesity.

## Figures and Tables

**Table 1:** Mothers’ Report of Their Child’s Food and Beverage Consumption on Weekdays and Weekends.

Foods and Beverages	Weekdays	Weekends
	*M* (*SD*)	*M* (*SD*)
Chips	2.5 (0.8)	2.8 (0.8) ***
Fast food	2.1 (0.8)	2.6 (0.8) ***
Fried foods	2.3 (0.8)	2.7 (0.8) ***
Fruit (not including juice)	3.8 (0.9)	3.8 (1.0)
Pizza	2.7 (0.8)	3.1 (0.7) ***
Sweet snacks/desserts	2.7 (0.8)	3.0 (0.9) ***
Vegetables	3.9 (1.0) **	3.7 (1.0)
Whole grains	3.7 (1.0) *	3.6 (1.0)
Plain water	4.2 (0.9) **	4.1 (1.0)
Soda/pop	2.1 (0.9)	2.3 (1.0) *

Note: *N* = 192. Frequency of consumption was measured on a 5-point scale, where 1 = never and 5 = always. Participants were provided several examples of items in these categories. Weekdays were defined as Monday through Friday afternoon and weekends as Friday evening through Sunday. *** *p* < 0.001; ** *p* < 0.01; * *p* < 0.05.

**Table 2:** Mothers’ Report of Their Child’s Portion Sizes on Weekdays and Weekends.

Foods and Beverages	Larger Portions on Weekdays	Larger Portions on Weekends	Same/Equal Portions	Does Not Consume
	%	%	%	%
Chips	3.1	38.5 ***	46.9	11.5
Fast food	7.8	45.3 ***	32.8	14.1
Fried foods	5.2	37.5 ***	49.0	8.3
Fruit	14.1	8.9	75.5	1.6
Pizza	6.3	45.8 ***	45.8	2.1
Sweet snacks/desserts	2.6	46.4 ***	44.8	6.3
Vegetables	17.7 ***	5.2	73.4	3.6
Whole grains	12.0 *	4.7	75.0	8.3
Plain water	6.8	7.3	83.9	2.1
Soda/pop	2.1	35.4 ***	32.3	30.2

Note: *N* = 192. Frequencies exclude mothers who indicated that their child did not consume the food or beverage item. *** *p* < 0.001; * *p* < 0.05.

**Table 3:** Mothers’ Perceptions of Foods and Beverages as Weekday or Weekend Items.

Foods and Beverages	Weekday	Weekend	Same/Equal
	%	%	%
Chips	2.6	40.1 ***	57.3
Fast food	10.4	55.7 ***	33.9
Fried food	6.3	44.8 ***	49.0
Fruit	6.8	4.2	89.1
Pizza	6.8	53.1 ***	40.1
Sweet snacks/desserts	4.7	41.7 ***	53.6
Vegetables	7.8	4.2	88.0
Whole grains	8.3	4.2	87.5
Plain water	5.2	2.6	92.2
Soda/pop	3.6	56.3 ***	40.1

Note: *N* = 192. *** *p* < 0.001.

**Table 4:** Mothers’ Feeding Goals on Weekdays and Weekends.

Goals	Weekdays	Weekends
	*M* (*SD*)	*M* (*SD*)
Health	4.01 (0.70)	3.89 (0.75) ***
Price	3.69 (0.90)	3.57 (0.95) **

Note: *N* = 192. Health = Subscale from the Food Choice Questionnaire, higher scores indicate more health-oriented; Price = Subscale from the Food Choice Questionnaire, higher scores indicate more price sensitive. *** *p* < 0.001; ** *p* < 0.01.
